# Rapid Progression of Molar Pregnancy to Choriocarcinoma With Pulmonary Metastases

**DOI:** 10.7759/cureus.83452

**Published:** 2025-05-04

**Authors:** Lucille J Wilkinson, Jared M Saifman, Pamela P Carbiener, Kelly L Molpus, Ruby A Deveras

**Affiliations:** 1 Obstetrics and Gynecology, Florida State University College of Medicine, Daytona Beach, USA; 2 Obstetrics and Gynecology, Halifax Health Medical Center, Daytona Beach, USA; 3 Gynecologic Oncology, Halifax Health Medical Center, Daytona Beach, USA; 4 Oncology, Halifax Health Medical Center, Daytona Beach, USA

**Keywords:** choriocarcinoma, gestational trophoblastic disease, gestational trophoblastic neoplasia, hydatidiform mole, molar pregnancy

## Abstract

Postmolar choriocarcinoma is a rare, highly malignant tumor of the placenta with characteristic histologic and clinical features. We present a case of a 31-year-old G3P2 female patient with evidence of a complete molar pregnancy indicated by symptoms of severe nausea and vomiting, a heterogenous, cystic mass on ultrasound, and human chorionic gonadotropin (hCG) levels above 900,000 IU/mL. Two days after the evacuation of the molar pregnancy, she developed symptomatic pulmonary metastases with markedly increased hCG levels and worsening symptoms. She was treated for pulmonary emboli and shortly started on EMA/CO (etoposide, methotrexate, actinomycin D, cyclophosphamide, vincristine) chemotherapy. However, due to persistently elevated hCG levels and rapid disease progression, she was ultimately referred to a tertiary trophoblastic disease center. She completed her chemotherapy regimen with subsequent return of her hCG levels to normal values and underwent a hysterectomy with complete removal of the tumor. While choriocarcinoma has an excellent prognosis, the accelerated progression and extreme hCG levels of this case highlight the need for further investigations into the management of molar pregnancy patients with high-risk features for gestational trophoblastic neoplasia (GTN), as well as the pathomechanism of this disease trajectory.

## Introduction

Gestational trophoblastic disease (GTD) encompasses a spectrum of placental lesions, including hydatidiform moles, characterized by abnormal trophoblastic proliferation. Gestational trophoblastic neoplasia (GTN) refers to a group of malignant trophoblastic tumors, which include invasive moles, choriocarcinoma, placental site trophoblastic tumor (PSTT), and epithelioid trophoblastic tumor (ETT).

A complete mole results from the fertilization of an enucleated ovum by one sperm, thus producing a 46XX zygote through the duplication or dispermic fertilization of the paternal genome [1,2]. This condition manifests as a large, edematous “hydropic” collection of tissue characterized by a proliferation of abnormal chorionic villi without fetal development. Clinical features of a complete mole can include vaginal bleeding, uterine enlargement disproportional to gestational age, hyperemesis gravidarum, new-onset hyperthyroidism, bilateral theca lutein cysts, and early-onset preeclampsia. These clinical manifestations commonly present in the second trimester. However, a complete mole is often diagnosed in the first trimester based on a “snowstorm” pattern on ultrasound and markedly elevated human chorionic gonadotropin (hCG) levels. Treatment involves prompt surgical uterine evacuation or hysterectomy, depending on the patient’s future childbearing goals and risk factors for GTN. The standard of post-evacuation care includes weekly monitoring of hCG until levels become, and subsequently remain, undetectable.

Choriocarcinoma is a rare, highly malignant tumor arising from invasive, rapidly proliferating cytotrophoblasts and syncytiotrophoblasts identified by anaplastic features and the absence of chorionic villi on histology. Fifty percent of GTNs occur after a molar pregnancy, 25% follow a pregnancy loss or tubal pregnancy, and the remaining 25% occur after a term or preterm pregnancy [3]. The risk of GTN after a complete molar pregnancy is approximately 15%, compared to a 5% risk after a partial molar pregnancy [4]. Risk factors for developing GTN after a molar pregnancy include uterine size larger than expected for gestational age prior to evacuation, hCG levels greater than 100,000 IU/mL, and the presence of bilateral theca lutein cysts [5].

Elevated hCG is the hallmark clinical indicator of GTN after a molar pregnancy. Patients may also present with abnormal uterine bleeding, pelvic pain, or symptoms linked to hCG stimulation effects, such as hyperthyroidism, theca lutein cysts, hyperemesis gravidarum, or preeclampsia. Furthermore, choriocarcinoma is usually metastatic at diagnosis, most commonly involving the lungs, vagina, central nervous system, or liver. Symptoms of metastases include dyspnea, cough, chest pain, and hemoptysis when associated with the lungs, vaginal bleeding or purulent discharge when associated with the vagina, and headache, neuropathy, dizziness, nausea, visual disturbances, and/or hemiparesis when associated with the brain. GTN can occur weeks to years after the antecedent pregnancy. However, it most commonly occurs in the surveillance period after a molar pregnancy. It is most often diagnosed via abnormal hCG levels, rather than symptoms, when a molar pregnancy is being followed post-evacuation. The International Federation of Gynecology and Obstetrics (FIGO) diagnostic criteria for postmolar GTN include (1) a plateau of hCG across four measurements over a period of three weeks or longer, (2) a rise in hCG for three consecutive weekly measurements over a period of at least two weeks, or (3) histologic confirmation of choriocarcinoma [6].

We present a case describing a 31-year-old woman who rapidly progressed from a complete molar pregnancy to choriocarcinoma with symptomatic pulmonary metastases within the span of 22 days. The objective of the case report is to highlight the interesting and unusual features of this patient's diagnosis and clinical course, to help better understand the GTD and GTN spectrum and its management. The patient provided informed consent, and patient anonymity was preserved.

## Case presentation

**Figure 1:**
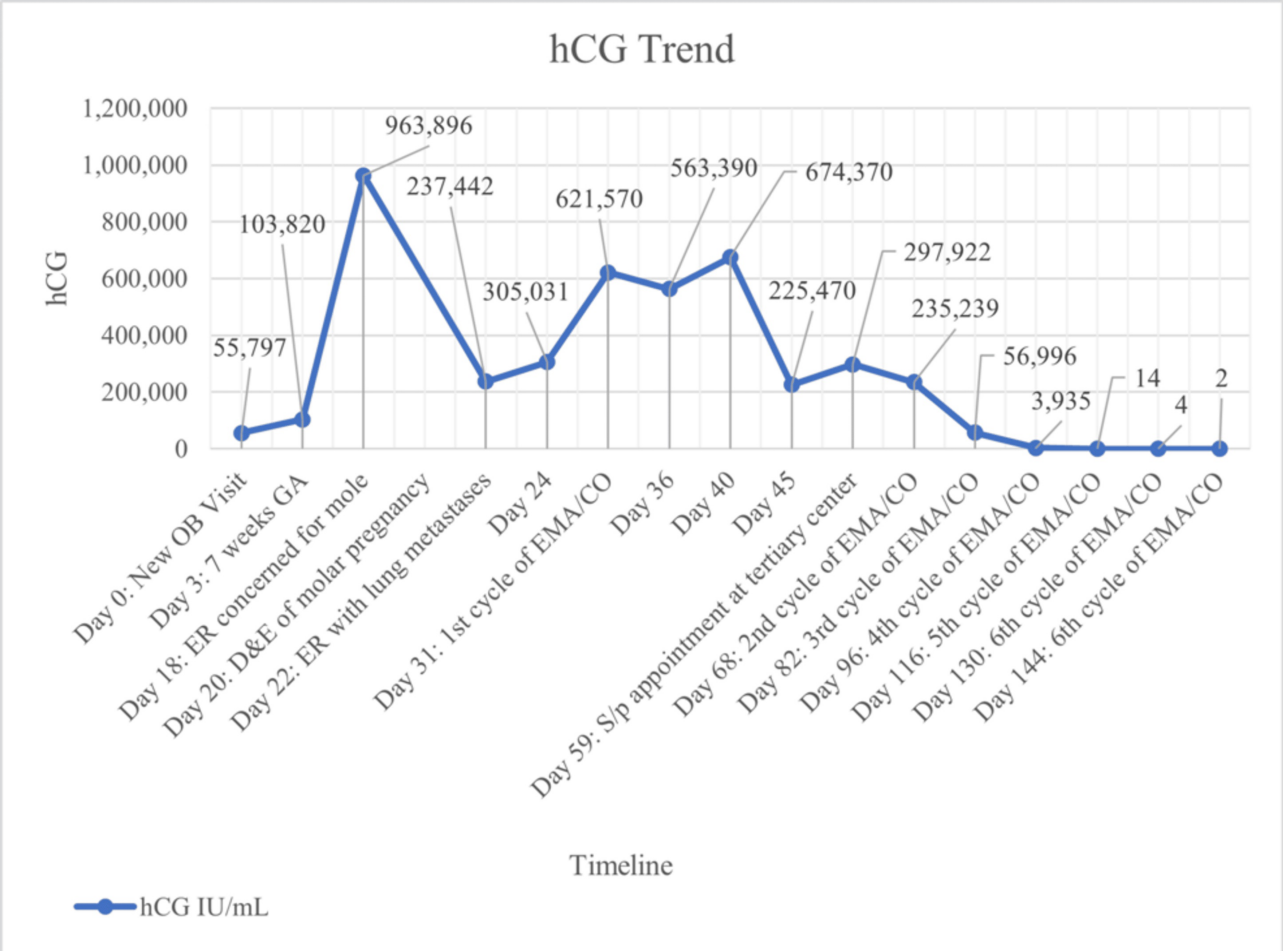
hCG trend hCG: human chorionic gonadotropin; OB: obstetrical; GA: gestational age; ER: emergency room; D&E: dilation and evacuation; EMA/CO: etoposide, methotrexate, actinomycin D, cyclophosphamide, vincristine; S/p: status post

The patient is a 31-year-old G3P2 of European descent who presented to the outpatient OBGYN office for a new OB visit on day 0. The patient was in overall excellent health with a history of two full-term, uncomplicated pregnancies and no chronic or systemic illnesses. The patient was a former smoker. In-office transvaginal ultrasound did not demonstrate a fetal pole and noted an irregularly shaped gestational sac measuring six weeks 0 days. The patient was scheduled to return to the office in two weeks to review the hCG trend and obtain an ultrasound. hCG obtained later that day was 55,797 IU/mL. On day 3, hCG was 103,820 IU/mL. The hCG trend throughout the patient’s presentation is shown in Figure 1. hCG levels often range between 100 IU/mL and 100,000 IU/mL for GTD and GTN, with a few cases rising to levels close to 1,000,000 IU/mL.

On day 18, the patient presented to the emergency room (ER) after a non-medical (“sneak peek”) ultrasound site reported no heartbeat. She described extreme nausea and vomiting with a 36-pound weight loss since day 0. The patient denied vaginal bleeding and cramping. Vitals were blood pressure 152/98 mmHg, pulse 86 beats per minute, respiratory rate 18 breaths per minute, temperature 98.2 degrees Fahrenheit oral, and oxygen saturation 98%. Transvaginal ultrasound in the ER showed a 13 cm uterus containing a large, heterogeneous mass in the endometrium with innumerable small cystic structures. Chest X-ray showed no acute process. hCG was 963,896 IU/mL. CBC and comprehensive metabolic panel (CMP) were unremarkable. Thyroid-stimulating hormone (TSH) was 0.008 IU/mL, and free T4 was 5.52 ng/dL. Lab findings are shown in Table 1. The patient was discharged with a diagnosis of molar pregnancy and was instructed to follow up with her provider immediately.

**Table 1:** Lab values * indicates abnormal value. WBC: white blood cell; RBC: red blood cell; MCV: mean corpuscular volume; BUN: blood urea nitrogen; AST: aspartate aminotransferase; ALT: alanine aminotransferase; eGFR: estimated glomerular filtration rate; TSH: thyroid-stimulating hormone; hCG: human chorionic gonadotropin

Parameter	Value	Reference range
WBC	11.94*	5.40-10.80 × 10^3^/μL
RBC	4.84	3.79-5.19 × 10^6^/μL
Hemoglobin	14.3	11.2-15.7 g/dL
Hematocrit	41.8	34.1%-44.9%
MCV	86.4	83.7-99.5 fL
Platelet count	202	126-432 × 10^3^/μL
Sodium	137	136-145 mmol/L
Potassium	4.0	3.5-5.1 mmol/L
Chloride	103	98-107 mmol/L
Carbon dioxide	21.6*	22-29 mmol/L
Anion gap	12	3-20 mmol/L
BUN	11.1	6-20 mg/dL
Creatine	0.63	0.51-0.95 mg/dL
Glucose	89	70-99 mg/dL
Calcium	9.7	8.6-10 mg/dL
AST	53*	10-35 U/L
ALT	47*	10-35 U/L
Alkaline phosphate	89	35-104 U/L
eGFR	121.8	mL/min
TSH	0.008*	0.27-4.20 μIU/mL
T4, free	5.52*	0.93-1.70 ng/dL
Beta-hCG quantitative	963,896*	IU/mL

On day 20, the patient presented to her OBGYN office with symptoms of severe nausea, vomiting, and bloating with no vaginal bleeding or cramping. Repeat transvaginal ultrasound in the office was consistent with findings in the ER, showing a heterogeneous mass with both cystic and solid components seen within the uterine body, a uterus measuring 13 cm, and no gestational sac or fetal pole seen. The right ovary was visualized and was within normal limits. The left ovary was not able to be visualized due to overlying bowel and gas. The patient was scheduled for same-day dilation and evacuation (D&E) for molar pregnancy.

Later that day, the patient underwent an unremarkable suction and sharp curettage. Samples were sent to pathology. Morning hCG was “greater than 200,000 IU/mL.” The patient was discharged the next day with instructions to return in three days to the outpatient office for repeat hCG, contraception management, and lung CT order due to the rapid trajectory and magnitude of the hCG levels.

On day 22, the patient presented to the ER with symptoms of tachycardia, elevated blood pressure, and shortness of breath. CT chest angiogram showed a small non-occlusive segmental to subsegmental pulmonary embolism in the right lower lobe without right heart strain and pulmonary edema with small bilateral pleural effusions. Doppler ultrasound of the lower extremity was negative for deep vein thrombosis. Chest X-ray showed no acute infiltrates. hCG was 237,442 IU/mL. The patient was treated for a presumed trophoblastic pulmonary embolism and was discharged in two days on Eliquis with instructions for prompt follow-up with oncology and OBGYN.

On day 24, the same day as discharge from the hospital, the patient presented to the OBGYN office. The patient stated she felt fatigued and “wiped out.” The patient was instructed to remain on Eliquis. CBC, hCG, and CT chest were ordered to be repeated with a follow-up appointment scheduled to review the results. The patient was scheduled to see an oncologist and a pulmonologist. On day 24, hCG was 305,031 IU/mL, which was a 28% rise since the last measurement two days prior.

**Figure 2:**
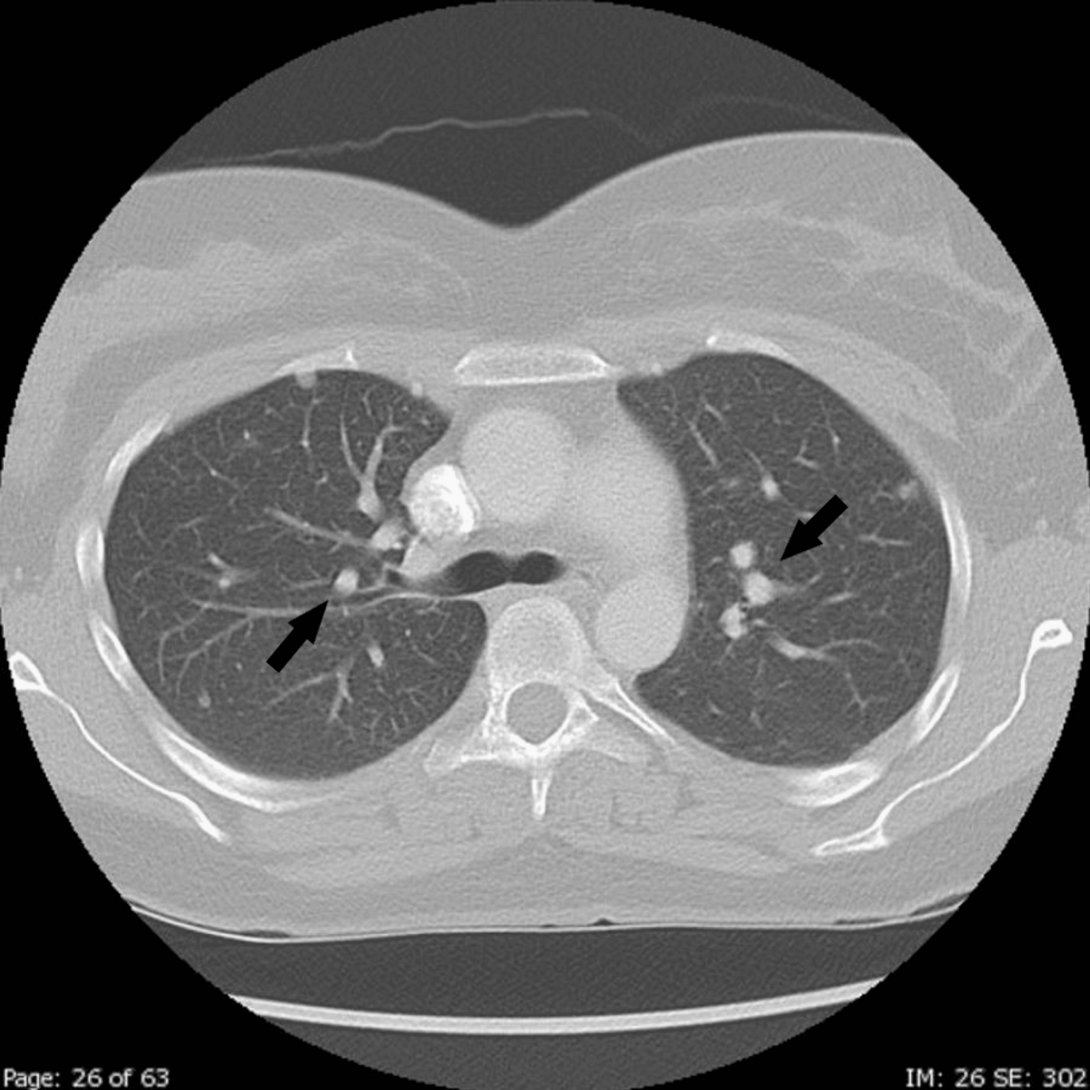
CT showing multiple metastatic lung nodules (black arrows)

On day 26, CT pulmonary angiogram showed multiple, bilateral non-calcified nodules ranging in size from 2 to 8 mm, as shown in Figure 2. The report indicated that these findings were highly suggestive of metastatic disease until proven otherwise. No acute focal pulmonary infiltrate or pulmonary vascular congestion was noted, and the scan was negative for pulmonary embolism.

On day 27, the patient returned to the OBGYN office to review the results. The repeat CT angiogram (CTA) did not suggest any emboli after five days of anticoagulation, but the multiple nodules were concerning for metastatic disease. The rise in hCG was also highly concerning.

**Figure 3:**
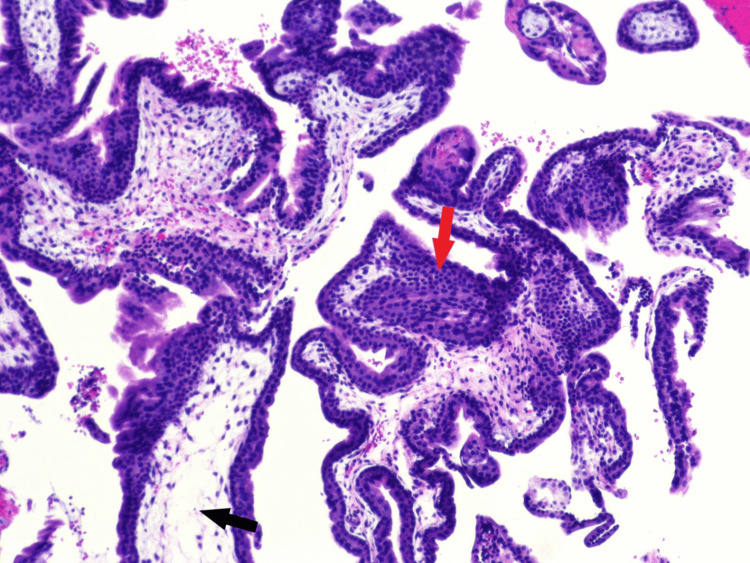
Pathology slides from D&E showing trophoblastic proliferation (red arrow) and placenta villi with hydropic degeneration (black arrow), consistent with hydatidiform mole D&E: dilation and evacuation

On day 31, the pathology report from the D&E on day 20 was reported, which indicated placental villi with hydropic degeneration, cistern formation, and trophoblastic proliferation, morphologically consistent with complete hydatidiform mole, as shown in Figure 3. The villous tissue was diploid and negative for P57, consistent with a complete mole.

On day 31, the patient presented to the obstetrician with worsening fatigue, persistent shortness of breath, nausea, bloating, and abdominal discomfort with no vaginal bleeding. A transvaginal ultrasound in the office revealed rapid regrowth of tissue. The pulmonologist was unable to obtain histologic confirmation of pulmonary nodules due to their size. The consensus was to proceed with aggressive and expedient intervention. The patient was admitted to the hospital that day to obtain repeat labs, PET scan, and MRI of the lung and brain in preparation for chemotherapy. MRI scan of the brain showed no evidence of brain metastasis. hCG remained “greater than 200,000 IU/mL,” and it was uncertain if lab analysis at the hospital was able to provide quantitative values greater than 200,000 IU/mL. A request was made to the lab to provide more specific titers if possible. The patient received the first cycle of EMA/CO (etoposide, methotrexate, actinomycin D, cyclophosphamide, vincristine) chemotherapy on the same day as admission.

On day 46, the patient was seen for follow-up in the outpatient OBGYN office. The patient reported symptoms of nausea and vomiting after the first cycle of chemotherapy. A transvaginal ultrasound in the office revealed an 8 x 10 cm mass, similar to the previous ultrasound, with cystic components consistent with a complete mole. Findings indicated minimal regression, but no progression of tissue volume in the uterus. hCG from day 45 was 225,470 IU/mL, a less-than-optimal decrease in hCG. Conversations ensued with the oncology team and pathology consultants regarding the perplexing nature of the case and the concern about the disease being refractory to chemotherapy. The patient was scheduled for a second opinion at a trophoblastic disease center.

On day 52, the patient was seen at a trophoblastic disease center. Given her partial response to first-line chemotherapy, her pathology was reevaluated. The case was sent to Brigham/Massachusetts General Hospital with a final report of abnormal immature chorionic villi and abundant, very atypical clusters of biphasic trophoblasts. Additional pathology will be obtained at the completion of her chemotherapy. The patient was diagnosed with stage 3 postmolar choriocarcinoma with multiple sub-8 mm lung metastases and WHO scoring of 5 (4 for hCG levels and 1 for lung metastases sites). Discussions ensued regarding the continuation of EMA/CO versus de-escalation to trialing weekly methotrexate. The patient wished to proceed with a single agent to avoid toxicity with EMA/CO. Unfortunately, hCG continued to climb, and she was switched back to EMA/CO. Over the next four months, the patient underwent six more cycles of EMA/CO with subsequent normalization of her hCG. The patient eventually underwent a hysterectomy with pathology confirming complete removal of the neoplasm.

## Discussion

This case highlights a rare and perplexing presentation of a postmolar choriocarcinoma with extremely rapid progression and significant elevations in hCG. Notably, this patient did not present with typical risk factors for choriocarcinoma, such as extremes of age or Asian ethnicity [7,8].

Risk factors for developing GTN after a molar pregnancy include pre-evacuation uterine size larger than gestational dates, hCG levels exceeding 100,000 IU/mL, and bilateral theca lutein cysts [5]. Upon the diagnosis of a molar pregnancy, this patient demonstrated significant risk factors, including a hCG of 963,896 IU/mL and a uterus measuring 13 cm at eight weeks and four days gestation, though no theca lutein cysts were noted on ultrasound. Current FIGO guidelines recommend prophylactic administration of methotrexate or actinomycin D chemotherapy for patients with high-risk features immediately following molar evacuation [6,9]. This has been shown to reduce the incidence of postmolar GTN by 3%-8% [10]. However, this patient developed pulmonary metastases just two days post-evacuation, raising questions about whether prophylactic chemotherapy would have slowed the progression of the tumor, changed the prognosis or outcome, caused unnecessary side effects, or rendered ineffective due to chemotherapy resistance.

The timeline of this case also underscores the gaps in current recommendations for managing patients with high-risk molar pregnancies. The standard of care after a molar pregnancy is to obtain weekly hCG levels until levels remain undetectable. However, this patient presented with pulmonary metastatic symptoms confirmed by subsequent imaging two days after the evacuation, well before she was due to receive her first hCG check. Additionally, the FIGO diagnostic criteria of a postmolar GTN include a hCG plateau over a three-week period and a greater than 10% increase of hCG over two weeks [6]. By the time the patient met the FIGO diagnostic criteria - notably the patient had a 161% increase in hCG over three values in two weeks - she had already received her first cycle of chemotherapy. This potential delay per guidelines highlights a need for earlier and more intensive monitoring of patients with high-risk features after a molar pregnancy.

In most cases of choriocarcinoma, hCG levels range between 100 and 100,000 IU/mL [11]. However, this patient presented with a hCG of 963,896 IU/mL upon initial diagnosis of a molar pregnancy, followed by a hCG of 237,442 IU/mL upon identification of pulmonary metastases. The hCG levels continued to increase to over 600,000 IU/mL before the EMA/CO treatments initiated a downward trend. These findings underscore the rapid and unanticipated progression of the disease. Difficulty quantifying trends was encountered due to the limits of the lab reports, which initially did not quantify any levels above 200,000 IU/mL. The inability to trend the hCG levels in real time was a potential disadvantage to managing this patient’s care. While more often than not hCG levels do not rise greater than 100,000 IU/mL, this presentation highlights the need to reevaluate how high-risk molar pregnancies are followed and the capabilities of the diagnostic testing.

There have been only a few other reported cases of such rapid progression of a complete molar pregnancy to choriocarcinoma; however, there is variability between the cases regarding the interval of time, type of molar pregnancy (complete vs. partial), and the subsequent type of GTN (choriocarcinoma vs. PSTT or ETT) [12-15]. In one case, a 29-year-old presented with irregular vaginal bleeding and hCG of 83,962 IU/mL with antecedent pregnancy of full-term cesarean section seven years prior. Imaging revealed myometrial invasion, and a diagnosis of choriocarcinoma was confirmed. Debate ensued over whether the GTN developed from the pregnancy seven years prior or if there was a new onset pregnancy that rapidly progressed to GTN [12]. Another case described choriocarcinoma during a term pregnancy in a patient with a prior history of molar pregnancy [13]. Other reports have described the rapid progression of molar pregnancies to invasive moles, which is another type of GTN. There are also reported cases of postmolar choriocarcinoma after intervals as long as seven years [14]. An additional report described a G1PO 20-year-old female patient with a prior history of a hydatidiform mole who developed respiratory distress and coma due to metastatic postmolar gestation neoplasm [15]. This patient did experience a delay in treatment due to the COVID pandemic. The resurgence of metastatic disease over a year later and the rapid clinical deterioration of this patient emphasize the aggressive nature of this pathology and the potential for severe complications. These variations of this rare disease highlight the perplexing nature of the GTN transformation and underscore the need for further investigation into the mechanisms driving such diverse disease trajectories.

The context of this case in a smaller community also shines a light on the management of rare pathologies in areas without specialized centers. Rare cases that require expedient treatment can present anywhere, including smaller hospital systems, calling into question how to deliver care to these patients and how to expand the accessibility of specialty services. The availability of gestational trophoblastic centers to call and give guidance to local community hospitals, where patients like this might show up, could allow for more expedient treatment and better outcomes for these patients. EMA/CO could be administered successfully in the community setting, and its prompt initiation is necessary to save a patient’s life in rapidly progressing cases, like this one. While this patient eventually traveled to a GTD center, there is a potential for this case to be taken care of locally with the help of telephone consults from GTD centers.

## Conclusions

This report describes a case of stage 3 choriocarcinoma with a WHO risk score of 5, which presented two days status post D&E of a complete molar pregnancy in a 31-year-old G3P2 female patient. Despite the typically good prognosis for choriocarcinoma, this case report was concerning for the rapid progression of tissue growth post D&E and initial poor response to chemotherapy, requiring full EMA/CO therapy and eventual hysterectomy. It raises questions regarding the management and timing of the treatment of patients with complete molar pregnancy with clinical risk factors for GTN. Furthermore, this case highlights the need to research more of the mechanisms at work with postmolar choriocarcinoma and to understand what risk factors may be at play, considering this patient did not have the studied risk factors for GTN. Additional investigations into the unique cell lines of GTN may provide individualized prognostic factors that modify monitoring and treatment.

While choriocarcinoma typically has a predictable progression and excellent prognosis, this case did not follow the typical course in presentation and response to treatment. This patient initially hoped to preserve fertility but was unable to do so. It is unlikely that earlier diagnosis and treatment would have allowed the preservation of fertility. This case highlights the challenges faced by practitioners, like identifying trends when reporting varies among labs, and the need for more research on the intricacies of this disease spectrum.
